# Management of Tracheal Diseases in Children

**DOI:** 10.3389/fped.2020.00297

**Published:** 2020-06-12

**Authors:** Nagarajan Muthialu, Madhavan Ramaswamy, Arun Beeman, Yi-Ting Yeh

**Affiliations:** Tracheal Team, Department of Cardiothoracic Surgery, Great Ormond Street Hospital, London, United Kingdom

**Keywords:** trachea, trachea—surgery airway obstruction—surgery, trachea abnormalities, tracheo bronchial, cardiopulmoanry bypass

## Abstract

Tracheal pathology in children are primarily congenital. They can be considered primary or *de novo*, when this is seen as an inherent defect within the cartilages of the tracheal segment. While segmental cartilage defects are very rare, there are often occasions when one or more cartilages can be considered missing from the length of trachea, contributing to airway abnormality. Secondary tracheal pathologies can often be seen in relation to disorders affecting nearby vascular elements or thoracic cage in general. In general, the pathological entity of tracheal disorders can be classified into either tracheomalacia or tracheal stenosis.

## Tracheomalacia

Tracheomalacia, or otherwise seen as weakness of tracheal wall can again be either primary or acquired.

Primary tracheomalacia can often be diffuse, with weakness affecting many cartilaginous arches, especially in the distal half of trachea. This is often seen in preterm, or in neonatal phase, with clinical presentation often needing ventilation to support the respiratory system in these small babies. Extensive nature of this weakness can sometimes extend beyond carina, leading to the entity “tracheobronchomalacia.”

Acquired tracheomalacia can often be secondary to extensive infection and inflammation, seen mainly following an episode of tracheitis, for example in croup or any similar viral infection.

Similarly, pathologies of nearby vascular tree, namely vascular rings and innominate artery compression syndromes, can often lead to localized area of tracheal compression and persistence of tracheal weakness, thereby causing tracheomalacia. This may persist for a very long period, even after relief of compressing elements, especially if such a relief had happened later in life.

## Evaluation of Children With Tracheomalacia

The main clinical feature of a child with tracheomalacia is often a noisy breathing, or stridor. Apart from this, the presentation is usually subtle. Only rarely we come across a situation wherein the child needs invasive ventilation due to severe respiratory distress related to this, and such a presentation is often seen in very small neonates or preterm babies.

The usual initial array of investigations often begins with computerized tomographic scan, which can reveal the quality of underlying pulmonary parenchyma in a child with respiratory distress. The main reason for a cross-sectional analysis is to assess the nearby vascular tree, to rule out causes that could potentially be treated—namely vascular ring and innominate artery compression syndrome.

Also, the cross-sectional study could reveal pathologies of thoracic inlet, including inlet narrowing and other abnormalities of cartilage and any segmentation defects in rare genetic syndromes.

Further evaluation includes bronchoscopy and bronchogram. While bronchoscopy can reveal the luminal assessment of airways and demonstrate lack of complete cartilaginous ring (see below), thereby ruling out complete tracheal stenosis, this often reveals localized narrowing in situations of vascular compression.

Bronchogram can be a very efficient dynamic investigation, revealing antero-posterior collapse, including the extent of collapse and severity of such change in respiratory cycle. This can be a diagnostic confirmatory test in many babies, often leading to making management plan.

Our practice include combining micro-laryngoscopy along with bronchoscopy and bronchogram thereby allowing for a complete assessment of entire airways, as the combined laryngomalacia and tracheomalacia is more often seen than mentioned.

Management of tracheomalacia depends on presence of correctable elements. As mentioned above, correctable vascular compression should be primarily attended to as appropriate.

Conservative management of tracheomalacia is very effective in many occasions. The medical management of these children is best by a multi-disciplinary team with input from many specialists contributing to various areas of improvement of care. This can include supportive measures such as non-invasive ventilation (NIV). Severe form of tracheobronchomalacia may require mechanical support of airways to deliver this NIV, either in the form of prolonged intubation or tracheostomy. But both are not without relevant risks and complications.

Airway stenting is gaining more attention and experience in many units. While traditional airway stenting had always involved metal stents, which invariably lead to complications later in life, use of bio-degradable stents (see below) is increasingly popular. These stents offer initial “proof of principle” approach for assessment, followed by further stenting wherever appropriate.

Surgery do remain as an important adjunct to management of persistent and at times complex tracheomalacia. These surgical procedures take different form, depending on the nature of malacia, as well as pathophysiological background to it.

Many centers often consider aortopexy as a favored surgical option, especially in persistent cases. This is done by both thoracoscopically and by anterior approach from mini-upper sternotomy (1, 2). This is often combined with a diagnostic intraoperative bronchoscopy to reveal the extent of opening of trachea on moving aorta anteriorly.

**Figure 1:**
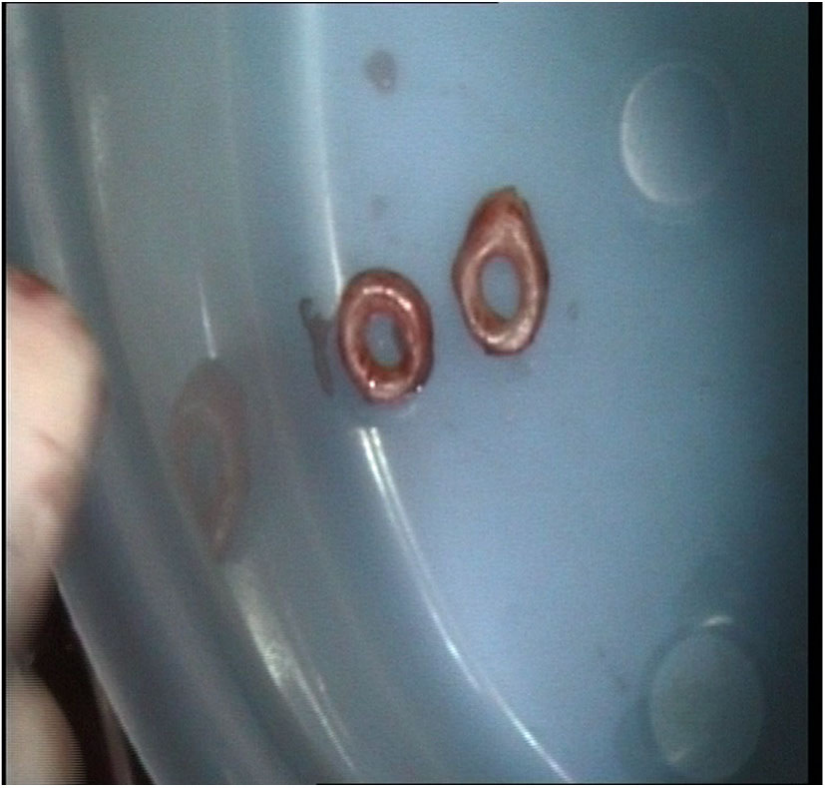
Picture demonstrating excised complete cartilaginous tracheal rings.

The indications for aortopexy remain unclear in these groups. Wherever there is a vascular element to presence of tracheal narrowing with/without tracheomalacia, aortopexy, by moving innominate artery and ascending aorta (Figure 1), offers better relief to the airway compression. But in certain conditions, including children following repair of trachea-esophageal fistula, aortopexy only offers indirect relief.

**Figure 2:**
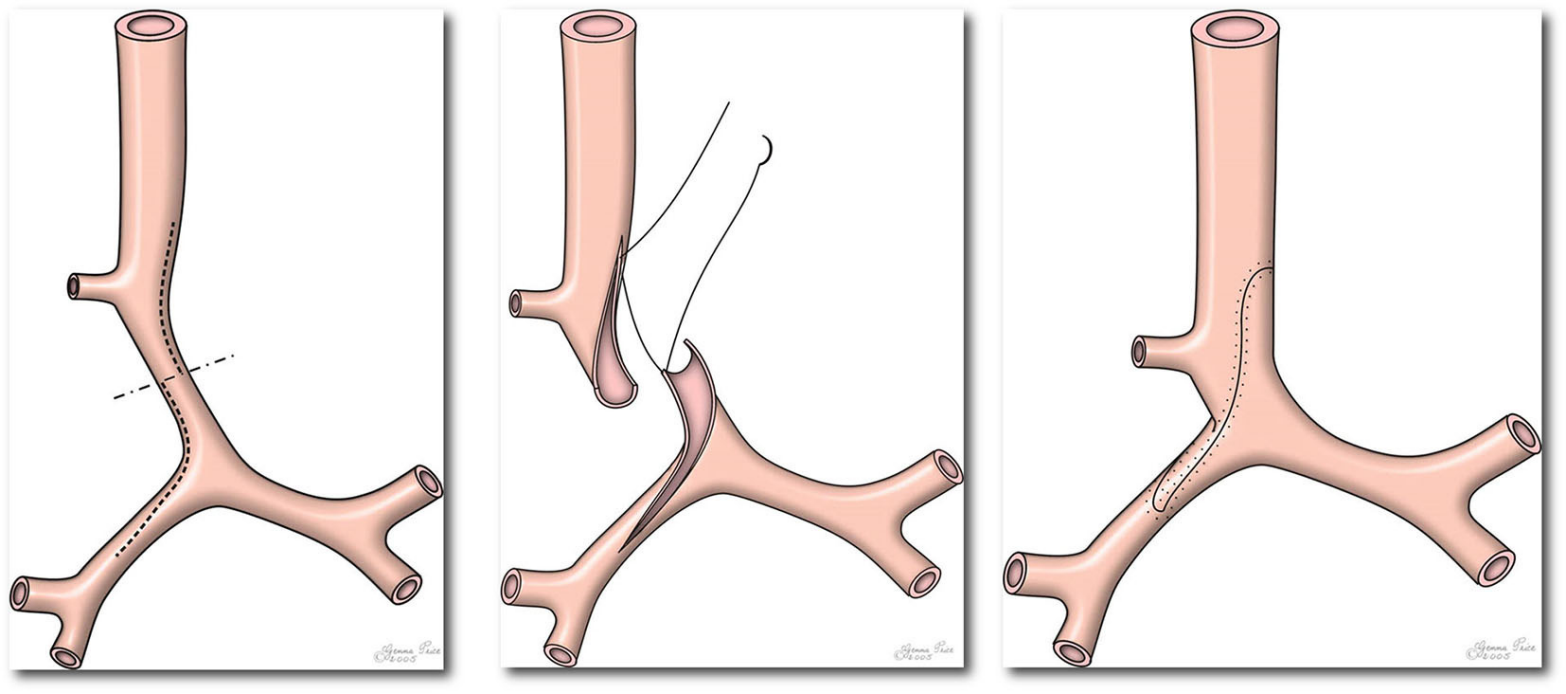
Picture demonstrating standard operating technique during slide tracheoplasty. Please note extension of incision across right main bronchus (Figure reproduced from Tsang V, Murday A, Gillbe C, Goldstraw P. Slide tracheoplasty for congenital funnel-shaped tracheal stenosis. *Ann Thorac Surg*. (1989) 48:632–5).

With advances in endoscopic procedures and relevant instruments, thoracoscopic aortopexy is also gaining popularity, offering similar results to the open method (Figure 2).

The main pathophysiology in this group remains as posterior prolapse of membranous part of trachea. In such situations, posterior tracheopexy offers better relief This involves fixing the membranous part of trachea to anterior spinal muscles, thereby keeping trachea open during most of respiratory cycles and avoiding spontaneous collapse during breathing. Early results are very promising, but further studies are required to fully understand the long term impact, especially on risk of aspiration in these children.

## Congenital Tracheal Stenosis

### Long Segment Congenital Tracheal Stenosis in Children

#### Background

**Figure 3:**
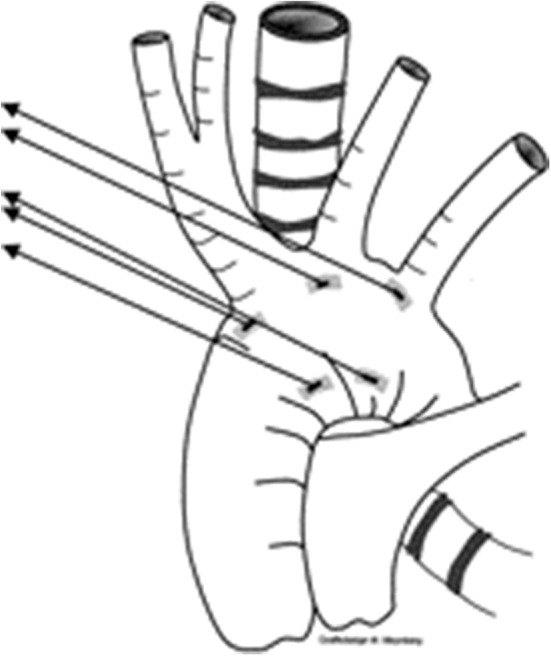
Picture demonstrating the position of sutures for aortopexy—one at base of innominate artery, and another at the distal end of ascending aorta.

Long segment congenital tracheal stenosis (LSCTS) is a rare congenital abnormality occurring in about 1 in 100,000 live births (3). In LSCTS more than 50% of the length of the trachea is stenosed, always due to complete tracheal rings (Figure 3). Over 70% of patients have associated cardiovascular abnormalities, and of them, two-thirds have left pulmonary artery sling (LPAS) (4). Symptoms relate not only to the length and severity of the narrowing but also to the associated cardiovascular abnormalities and the age at presentation. Symptoms range from respiratory failure soon after birth requiring extracorporeal membrane oxygenation (ECMO) support to mild to moderate severity like noisy breathing, respiratory distress, or recurrent chest infections. It can also remain asymptomatic especially in patients in whom it is incidentally diagnosed during elective intubation when they are evaluated for other abnormalities (5).

Until late 1980s, LSCTS was difficult to manage with treatment often considered unsuccessful. Various techniques were employed to enlarge the airway, the choice of which was dependent on the expertise of the core specialty of the managing surgeon. Some of the techniques during that period included autograft repair by Carl Backer in Chicago (6), pericardial patch repair by Robin Cotton in Cincinnati (7), and slide tracheoplasty by Victor Tsang, while Grillo (8) reported similar experience in adults. At Great Ormond Street Hospital (GOSH) in London all these techniques were used until slide tracheoplasty was considered to be the surgery of choice for treating LSCTS (9). Then in the year 2000, a multidisciplinary tracheal team and a structured clinical pathway were established, as treatment prior to that year was often driven by the specialist to whom the patient was referred with no clear management pathway often resulting in unsatisfactory outcomes. As part of this all patients referred with LSCTS were discussed in the departmental multidisciplinary meeting to decide their clinical management and clinicians involved were trained to improve their essential skills including fiberoptic and interventional bronchoscopy procedures. With this approach it was possible to demonstrate improved results, decreased hospital stay and increased savings within a short span of time (10). Following this in the year 2006, it was possible to establish GOSH as the national center for tracheal service for children in the United Kingdom.

## Care Pathway for LSCTS at GOSH

### Investigations

Below are the key investigations necessary to assess the airways before embarking on a definitive management, either surgical or conservative.

#### Bronchoscopy and Bronchography

Bronchoscopy is essential in demonstrating the extent and severity of tracheal stenosis. This can be performed either by patient's bedside who is unstable and ventilated or in the bronchoscopy suite when clinically stable. Although the role of bronchography is considered to be controversial by some treatment centers, it can provide useful information about airway dynamics especially about distal malacia which is superior to computerized tomography (CT) imaging and important in predicting long-term outcomes.

#### Contrast Enhanced CT of Airways, Lungs, and Heart

Contrast enhanced CT is essential not only to obtain information about the airway anatomy but also about the relationship between airways and the neighboring vascular structures which are important for planning surgery. It is also possible to obtain three dimensional models of the airways using CT images which are helpful in education of patients, careers, and clinicians.

#### Echocardiography

Due to high incidence of associated cardiovascular abnormalities, echocardiography is an essential investigation to delineate cardiac structure and function.

#### Optical Coherence Tomography

This technique is combined with bronchoscopy and bronchography to confirm presence of complete cartilage rings in the trachea and also to assess the integrity of cartilage in the distal airways. The tiny size of probe is advantageous for it to be passed through the severely stenotic airway where even the smallest bronchoscope (2.2 mm) cannot be passed.

#### Genetics

Genetic studies are useful in genotyping this rare abnormality. Samples are routinely sent from patient's blood and excised tracheal tissue. This is important as we have seen several sets of twins with this condition although a specific genetic abnormality is yet to be identified (11).

### Surgery

LSCTS repair by slide tracheoplasty is carried out through median sternotomy on cardiopulmonary bypass (CPB). Simple cardiac abnormalities, especially simple shunts, left pulmonary artery sling, and vascular ring can be simultaneously repaired. Pulmonary artery sling is repaired by disconnecting left pulmonary artery from right pulmonary artery, oversewing the remnant, passing left pulmonary artery into left pleura, and reattaching left pulmonary artery to main pulmonary artery close to the ductal origin. This ensures that the left pulmonary artery is clear of the tracheal reconstruction.

The technical details of slide tracheoplasty is beyond the scope of this article and can be found elsewhere (9, 12). In essence, the trachea is mobilized throughout the length of the stenosis by dissecting close to it and pushing the recurrent laryngeal nerves laterally away from the trachea. We have learnt that simple division of thyroid isthmus by diathermy provides considerable mobilization of trachea thereby decreasing tension in the trachea when the tracheal repair is completed. Additional mobilization of trachea can be achieved by division of posterior pericardial reflection seen behind left atrium and by freeing carinal lymph nodes.

**Figure 4:**
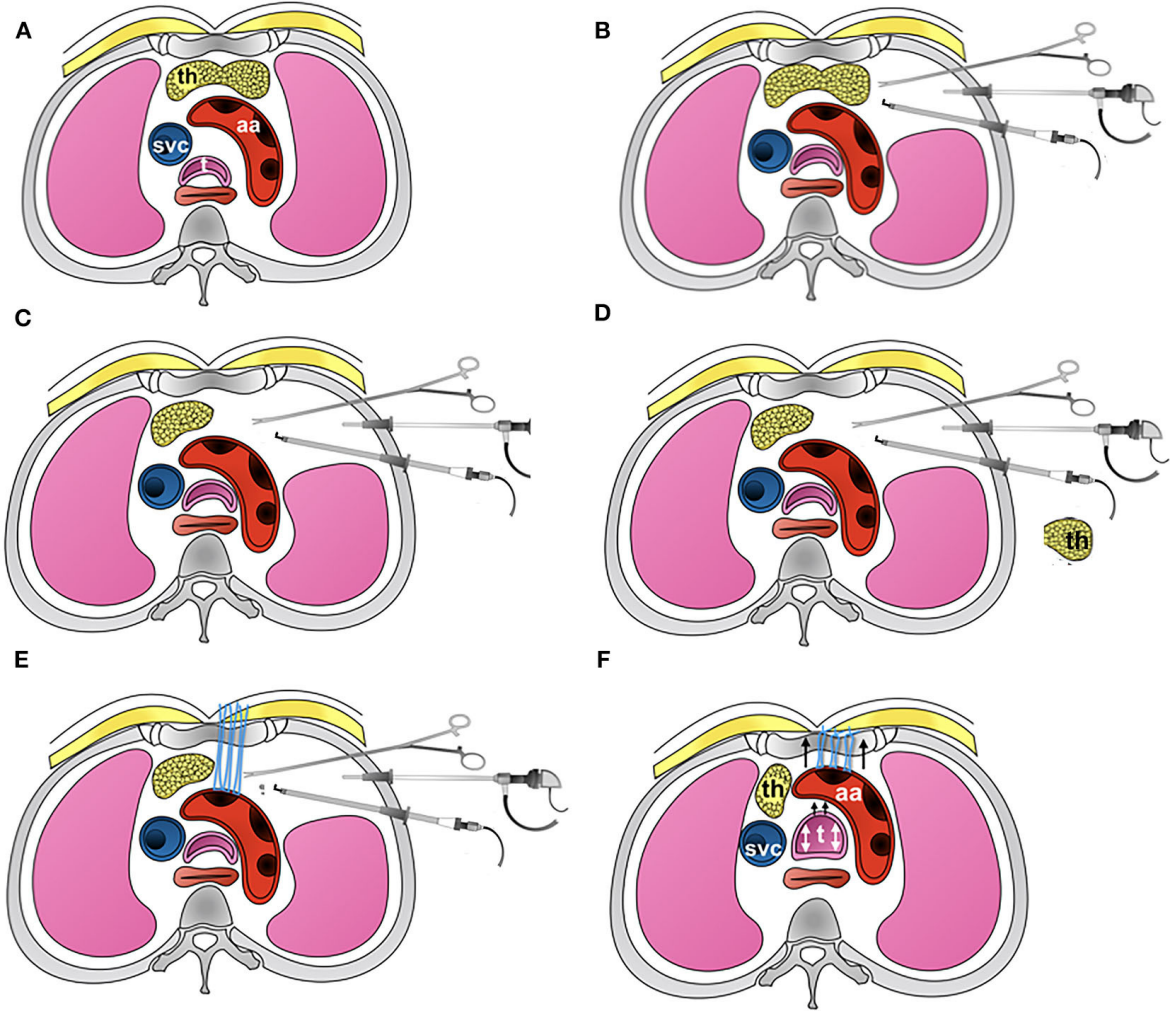
Picture showing the detailed steps involved in thoracoscopic aortopexy. **(A)** Standard axial anatomy at the level of aortic arch. **(B)** The three-port approach, showing three instruments in use for the aortopexy. **(C)** Shows left lobe of thymus having been removed. **(D)** Thymus is now removed away from the chest. **(E)** Application of sutures to adventitia at the proximal end of aortic arch, where the first branch, brachiocephalic artery, arises from. This in turn I brought out through the sternum. **(F)** The suture are tied, demonstrating relief to tracheal lumen when tied - additionally confirmed by bronchoscopy.

Trachea is then transected across the midpoint of stenosis and longitudinal incisions are made along cephalic segment and caudal segment, posteriorly, and anteriorly, respectively. The incision in the caudal segment is to be extended into the bronchus only if it is confirmed to be stenotic due to complete rings (Figure 4). After sliding, the two incised ends are then anastomosed by interrupted and everted 5:0 PDS sutures. Repair by continuous suture was abandoned as three patients early in our experience had tracheal dehiscence post-operatively. The repair site is sealed with TISSEEL fibrin glue once protamine has been given and heart decannulated. The repair site is always checked by fiberoptic bronchoscopy before weaning from CPB so that revisions can be promptly performed if necessary. The endotracheal tube is positioned at the center of the repair. Our practice is not to routinely extubate patients on the operating table as small amount of blood often enters the airway and it will be useful to have endotracheal tube in the early post-operative period for effective airway clearance. Routine prophylactic antibiotics are given post-operatively and risk of mediastinitis can be mitigated by irrigating the wound electively with dilute betadine for 48 h which has been our practice for the last 20 years.

### Postoperative Care

Ventilation support is gradually weaned over the first 24 h as possible unless there are reasons like an associated cardiac abnormality that was either operated simultaneously requiring continued cardiorespiratory support or if it was decided to repair it in stages, say few days apart, when the chest had to be left open occasionally. Bronchoscopy assessment is not routinely necessary prior to extubation as most of them can be successfully extubated in the early post-operative period unless the airway is severely malacic or the bronchus is stretched where long-term ventilation support may be required. Extubation can also be delayed in prematurity, small size of the patient, and presence of associated syndromic abnormalities. After extubation the patient is stepped down to the ward for further medical care. Airway is electively assessed by bronchoscopy in a week's time unless there are features of airway obstruction when an urgent evaluation becomes necessary. Granulation tissue, mild re-stenosis, and scarring of the airways can often be dealt successfully with balloon dilatation under fluoroscopy control. Removal of granulation tissue by forceps or laser are rarely necessary, the latter technique carries a risk of severe restenosis. It is worth mentioning that most of the difficulties encountered during ventilation support in the post-operative period can be managed by optimal positioning of endotracheal tube, effective clearance of airways with help from specialist physiotherapist, and by endoscopic intervention of airways.

### Outcomes

Our experience on slide tracheoplasty now goes to almost 200 children with LSTS having undergone surgical repair. Median age at surgery was 4 months (range: 0–50 months) and the median weight was 4 kg (range 1.2–38 kg). ECMO support was required preoperatively in 12% of patients. Median tracheal diameter was 2 mm and more than 90% of trachea was stenosed in 80% of patients. All patients had complete cartilage rings. Sixteen percent of patients had aberrant right upper lobe bronchus (*n* = 20), 12% had carinal trifurcation (*n* = 15), and 7% had single lung morphology (*n* = 9). Eighty percent of patients had associated cardiovascular abnormalities of which two-thirds had left pulmonary artery sling.

Outcomes following standard slide tracheoplasty is very satisfactory with actuarial survival post-repair being 88.4% (Table 1). In our experience, in the first decade of the series, survival was 80% at 110 months compared with 89.9% in the second decade.

**Table 1:** Actuarial survival on children undergoing slide tracheoplasty.

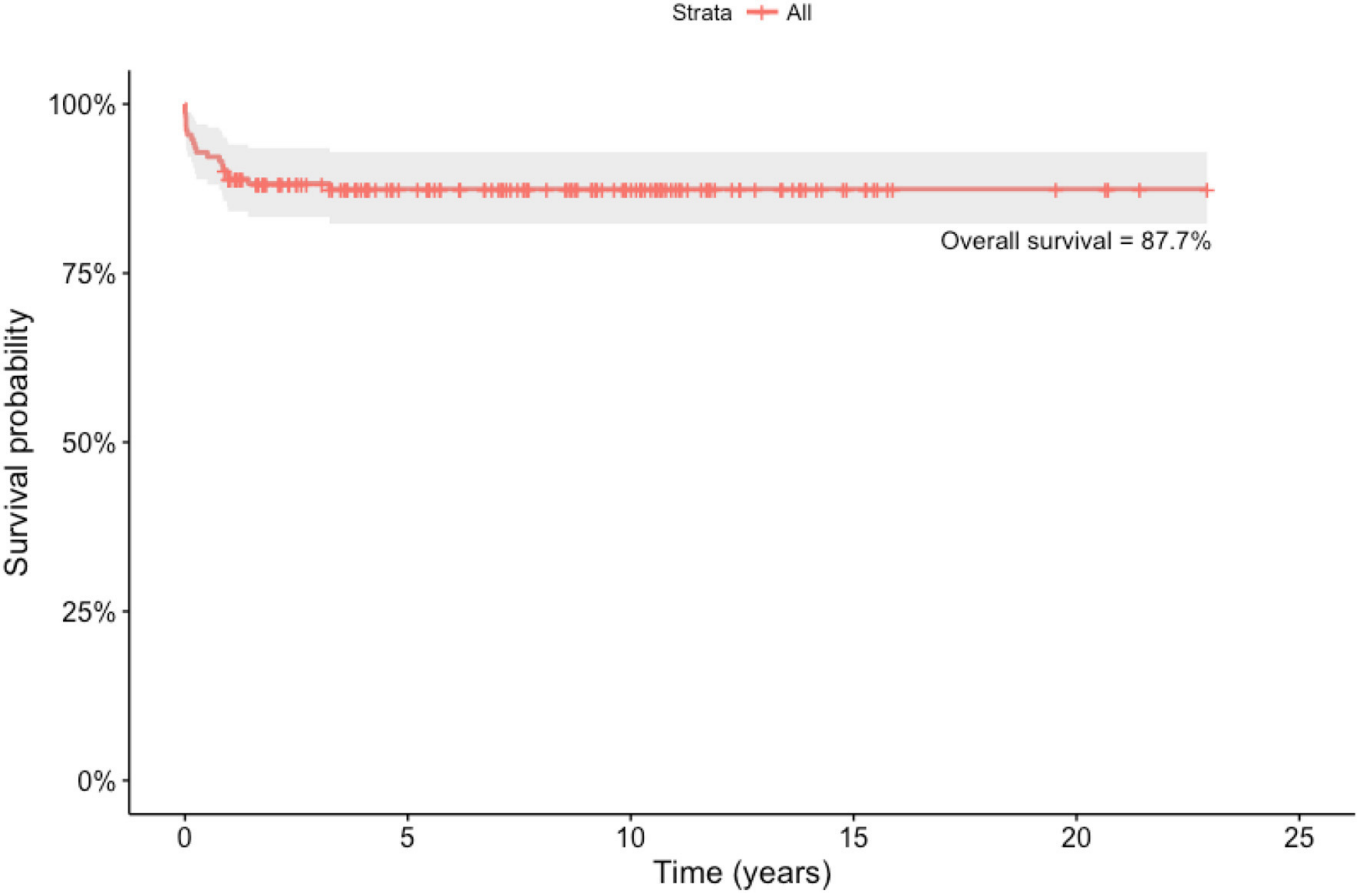

Table 2 shows the impact of preoperative bronchomalacia, demonstrating its ability to significantly and adversely affect survival (*p* < 0.001), 71.5% at 200 months as compared to 94.5% survival in patients without bronchomalacia. Bronchial stenosis was also significantly associated significantly with increased mortality with a survival of 74.8% as compared to 94.1% in its absence.

**Table 2:** Impact of pre-operative distal bronchomalacia on survival after slide tracheoplasty [the blueline indicating survival without preoperative malacia (median actuarial survival at 94.6%) while redline indicating survival with malacia (median actuarial survival 61.3%)].

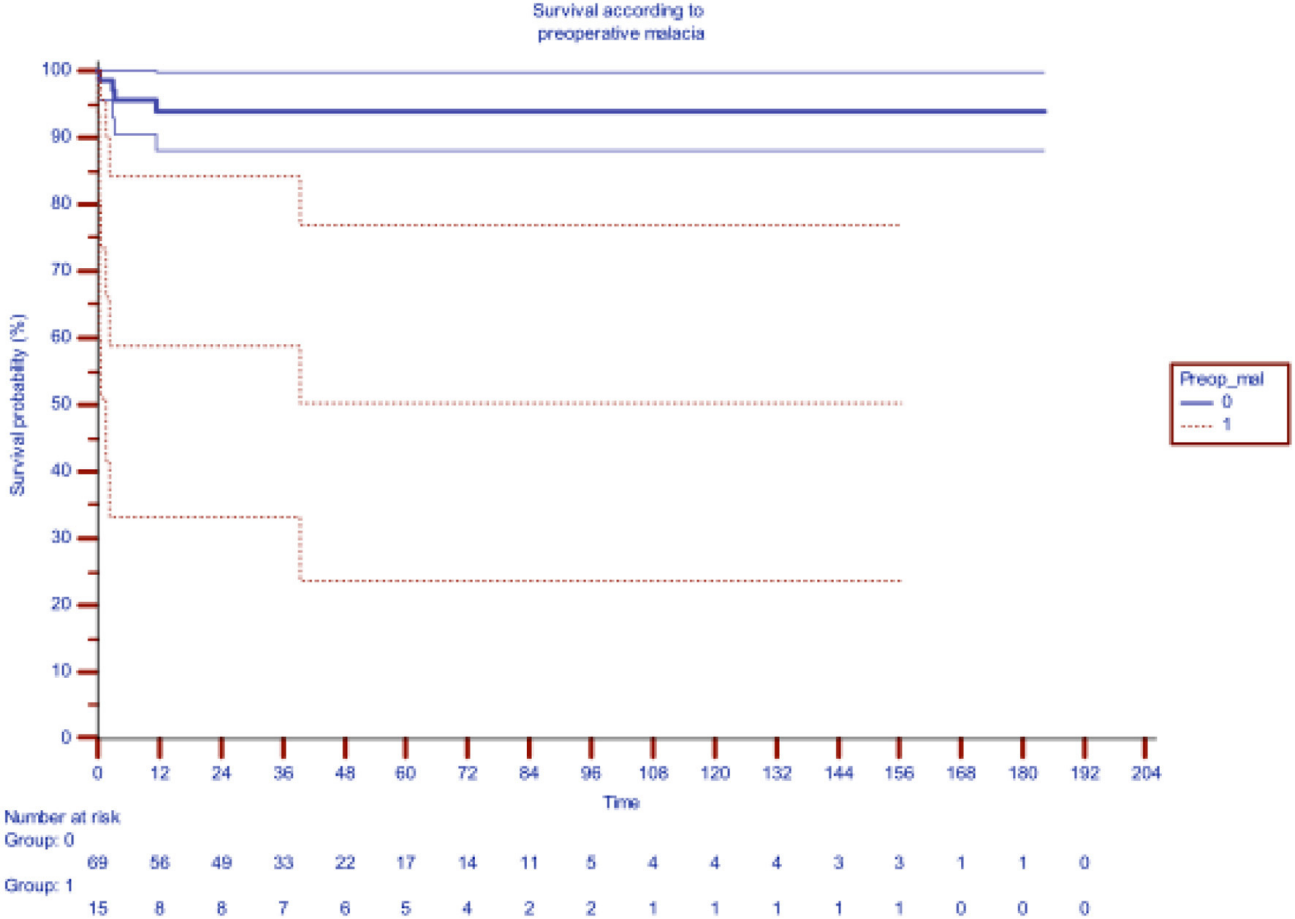

Although preoperative ventilation and ECMO support adversely affected mortality, only preoperative bronchomalacia and preoperative bronchial stenosis emerged as significant risk factors on multivariate analysis with a hazard function of 8.44 (*p* = 0.004) and 6.49 (0.011), respectively. Syndromic association, prematurity, and low birth weight are emerging as risk factors as we gain experience.

While association of cardiovascular defects make surgery more complex, there has not been any significant impact on overall survival in these children needing primary surgical repair of tracheal stenosis. Our management plan in these children include varying approach depending on the nature of symptoms: either primary airway or cardiac approach, especially if cardiac defects are considered too complex. otherwise, these children would go on to have combined cardiac correction at the same time as tracheal surgery.

Preoperative bronchomalacia was an important risk factor for requirement of airway stenting post-repair. Only 31% of those with bronchomalacia were stent-free at 120 months as compared to 82.6% of those without malacia during the same interval.

In the early years of our experience, stents used to treat airway malacia were metal, either balloon dilatable or self-expanding, only options available at that time. These metallic stents were faced with significant complications, with higher risk for erosion and in-stent stenosis. However, the risks were considerably mitigated with the use of biodegradable stents made of polydioxanone (PDS) material which are custom made to the patient (13). Airway integrity is well maintained for 2 months beyond which the biodegradable stents start to dissolve, completely by 3 months.

Although significant complications associated with metal stents have been mitigated with the use of biodegradable stents, they are associated with increased risk of airway granulations, treatable by serial balloon dilatations. Median number of postoperative balloon dilatations was two in our series (range: 0–27) of which 10 patients needed more than 10 dilatations.

Recurrent laryngeal nerve palsy was seen in 2.3% of patients (*n* = 3). No patients between the year 2000 to the year 2015 had mediastinitis with the usage of mediastinal irrigation. Although the median length of stay in the intensive care unit has fallen significantly over time to 13 days (range: 1–480 days, *p* < 0.05), the problem of managing complex patients still remains a major challenge.

Swallowing can be impaired in several patients postoperatively but it is seen to improve over time with most of them capable of feeding orally at the time of hospital discharge although a period of thickened feeds may be required (14). Detailed quality of life (QoL) assessment studied in thirty patients in our institution showed that the quality of life after slide tracheoplasty was similar to that of healthy children from perspective of both patients and parents (15). Also, patients with LSCTS having associated cardiovascular abnormalities did not have reduced quality of life compared to those without cardiovascular abnormalities but patients with syndromic associations, especially VACTERL association, had poor QoL scores.

### Comment

In the management of LSCTS, our experience have shown that use of slide tracheoplasty as the surgical technique of choice, a multidisciplinary team approach, and usage of biodegradable stents have achieved excellent outcomes including a good quality of life. The improvement in outcomes is also contributed by centralization of care which has reduced the cost of treatment and encouraged standardization of practice.

To conclude, long segment congenital tracheal stenosis is a rare congenital airway abnormality characterized by presence of complete cartilage rings in more that 50% of the length of trachea. It is usually associated with other congenital abnormalities especially cardiovascular abnormalities, left pulmonary artery sling being the commonest. Previously considered to be a fatal abnormality, the advent of slide tracheoplasty, a multidisciplinary team approach, and centralization of care had considerably improved the outcomes with an ability to achieve an excellent quality of life after surgery. There still remains a small subset of high-risk patients who are difficult to manage where case selection and alternative treatments may be required.

### Short Segment Stenosis

Short segment tracheal stenosis are quite rare, but can be present due to presence of complete cartilaginous rings in a localized region of trachea. Anatomical definition of such short segment stenosis varies in literature, with many considering “any stenosis that remains less than 50% of tracheal length” refers to short segment stenosis. Apart from the length of involvement, the clinical presentation can almost resemble that of LSTS, and so is not repeated under this section.

Traditional surgical approach remains surgical for symptomatic children. Surgery is carried out under cardiopulmonary bypass, for efficient management of physiology, while repair of trachea is carried out. This can be either resection with end-to-end anastomosis, or a modified short segment slide tracheoplasty. In either procedure, it is important the vascular arcade to trachea laterally is maintained for its integrity, to ensure adequate healing at anastomosis, as well as to prevent dehiscence. The risk of dehiscence is reportedly higher with resection of airways with end-to-end anastomosis, where a circular suture line is prone to vascular impingment in situations where lateral vascular arcade is interfered with, at surgery.

Occasionally isolated absence of cartilage rings, especially at juxta-carinal location can lead to the appearance of localized short segment stenosis. These isolated absence of cartilage rings are very unique embryologically and is prone for congenital absence of cartilaginous rings in these segments. The trifold of cartilage between distal trachea to left and right main bronchi are lacking, thereby leading to very short segmental stenosis of trachea.

Presentation is in early neonatal period due to severe respiratory distress often leading to supporting these children by ventilatory measures. Treatment is surgical, and involves primary resection of such a short segment stenosis and primary repair of distal trachea. Location of such stenosis close to carina makes this impossible to be treated by stenting, and complex involvement of bronchial origins can make this very difficult in overall management.

## Salvage of Trachea Following Various External Injuries Including Failure From Previous Surgery—Tracheal Reconstruction Measures

### Use of Autologous Pedicled Pericardial Patch

Autologous pedicled pericardial patch repair of trachea is a very versatile and sustainable technique used to reconstruct trachea mainly following previous surgical failure. This aims to support and salvage the airways with providing vascularized tissue to various areas, and is reproducible (16). Early results are very promising, and further study is required to demonstrate the ability of this vascularized tissue to reform itself within respiratory endothelium and further ciliary formation.

### Role of Tissue Engineering and Tracheal Transplantation in Children

Tracheal replacement for the treatment of end-stage airway disease remains a difficult challenge, severely limited by the difficulties around tissue engineering. The exact clinical application remains mysterious though compassionate use suggests this is a very valid and viable option.

While tissue engineering part with autologous stem cells and *in-vitro* modeling of cell population have gained excellent progress, there still seems to be a major hurdle around ideal tracheal conduit—with varying use from our center so far (17, 18). The last of such use involved cell seeding on a tracheal graft, previously decellularized—graft having been procured as part of lung transplant specimen from cadaver.

Such surgery is often considered salvage option for children with otherwise no possibility of airway repair and support, this is also considered major and very complex. often they need further support by stenting of reconstructed airways, with potentially long recuperation in hospital and community. Experience is limited to one center at the time of writing and further work is needed on many fronts including ethical aspects and progress in tissue engineering.

## Author Contributions

NM prepared the initial draft. Y-TY, MR, and AB contributed on the preparation of data and statistical analysis. Final edit was reviewed by all authors and approved before submission.

## Conflict of Interest

The authors declare that the research was conducted in the absence of any commercial or financial relationships that could be construed as a potential conflict of interest.
